# Ephemeroptera, Plecoptera, and Trichoptera on Isle Royale National Park, USA, compared to mainland species pool and size distribution

**DOI:** 10.3897/zookeys.532.6478

**Published:** 2015-11-05

**Authors:** R. Edward DeWalt, Eric J. South

**Affiliations:** 1 University of Illinois, Prairie Research Institute, Illinois Natural History Survey, 1816 S Oak St., Champaign, IL 61820, USA; 2 University of Illinois at Urbana-Champaign, Department of Entomology, 320 Morrill Hall, 505 S. Goodwin Ave., Urbana, IL 61801, USA

**Keywords:** Isle Royale National Park, Ephemeroptera, Plecoptera, Trichoptera, adult size, regional species pool

## Abstract

Extensive sampling for aquatic insects was conducted in the orders Ephemeroptera (mayflies), Plecoptera (stoneflies), and Trichoptera (caddisflies) (EPT) of Isle Royale National Park (ISRO), Michigan, United States of America, during summer 2013. The island was ice covered until 8,000 to 10,000 years ago and is isolated by 22–70 km distance from the mainland. Two hypotheses were examined: that ISRO EPT richness would be much reduced from the mainland, and that the species colonizing ISRO would be of smaller size than mainland, adults presumably using updrafts to bridge the distance from mainland sources. Data sets were developed for known mainland EPT species and size for those species. The first hypothesis was confirmed with the mainland species pool consisting of 417 EPT, while ISRO is known to support 73 species. Richness of EPT is directly related to the number of specimens examined. Small streams supported five EPT species, while 15–25 species were found in larger streams. Lakeshores had intermediate diversity. The second hypothesis was substantiated for stoneflies, but not for mayflies or caddisflies. Stoneflies apparently are poorer fliers than either of the other two orders.

## Introduction

**Figure 1.**
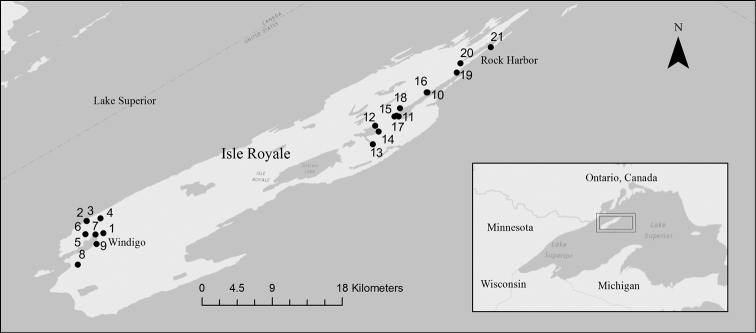
Sampling locations on Isle Royale National Park, Michigan, USA, during 2013. Points and numbers indicate sample locations as defined in Table 1.

Isle Royale National Park (ISRO) is an archipelago of islands located in cold, oligotrophic Lake Superior, Michigan, United States of America (USA). The main island is 72 km long and 14 km wide at its greatest dimensions (Kraft et al. 2010). It is presumed that all macroscopic life was eliminated from the island during the Wisconsinan glacial episodes. The region has been ice free for as much as 10,000 yr. Life repopulated by various means from mainland sources, a distance of 20–22 km from Minnesota, USA and Ontario, Canada or 70 km from the Keweenaw Peninsula of Michigan (Fig. 1). The entire park was assessed for natural resource condition within the past decade and much of what is known about the island and its fauna and flora is contained within Kraft et al. (2010).

**Table 1.** Locations sampled and dates visited to sample EPT taxa at Isle Royale National Park during 2013. Site numbers correspond to those used on Fig. 1. *Indicates incompletely sampled locations.

Site	Waterbody	Locality	Latitude	Longitude	6/17	6/18	6/19	6/20	7/27	7/28	7/29	7/30
1	Washington Cr.	1.6 km NE Windigo on Hugginin Tr.	47.92155	-89.14587	X		X					
2	L. Superior	Huginnin Cove CG	47.93499	-89.17524	X	X						
3	Huginnin Cr.	Huginnin Cove CG	47.93491	-89.17479	X	X						
4	Trib. L. Superior	Huginnin Cove Tr. E	47.93854	-89.15144		X	X					
5	Trib. L. Superior	Huginnin Cove Tr. W	47.91959	-89.17672			X					
6	Trib. L. Superior	Huginnin Cove Tr. W	47.91973	-89.17662			X					
7	Trib. L. Superior	Huginnin Cove Tr. W	47.91965	-89.15921			X					
8	Grace Cr.	Feldtman Lake Tr.	47.88451	-89.18843				X				
9	Trib. L. Superior	Feldtman Lake Tr.	47.90878	-89.15706				X				
10	L. Superior	Daisy Farm CG	48.09213	-88.59458					X		X	
11	L. Superior	Moskey Bay CG	48.06397	-88.64317					X			
12	*L. Richie	NE end at Richie Lake Tr.	48.05259	-88.68327						X		
13	*Outlet L. Richie	along Indian Portage Tr.	48.03133	-88.68655						X		
14	*Trib. L. Richie	along Indian Portage Tr.	48.04607	-88.67715						X		
15	Trib. L. Superior	0.6 km NW Moskey B. CG, L. Richie Tr.	48.06384	-88.65065							X	
16	Benson Cr.	Daisy Farm CG	48.09228	-88.59574							X	
17	Trib. L. Superior	0.3 km E Moskey B. CG at Rock Harbor Tr.	48.06463	-88.64669					X			
18	Trib. L. Superior	1.4 km E Moskey B. CG at Rock Harbor Tr.	48.07322	-88.64121					X		X	
19	Trib. L. Superior	Starvation Pt., Rock Harbor Tr.	48.11559	-88.54452								X
20	Tobin Cr.	at Mt. Franklin Tr.	48.12657	-88.53880								X
21	L. Superior	Rock Harbor (Snug Harbor)	48.14572	-88.48687								X

Little is known of the aquatic insects inhabiting the shores, streams, and lakes of ISRO (Bick et al. 1985). At least three ecological studies including aquatic insects have been conducted, but these involved sampling of larvae and genus level identification only, representing government literature and university theses that have not been published (Bowden 1981, Johnson 1980, Toczydlowski et al. 1979). A relatively few specimens of aquatic insects are known from regional museums (DeWalt unpubl. data).

The isolated nature of ISRO and lack of roads on the island make it logistically difficult to inventory. The island may be reached by ferry, personal watercraft, or by plane. It is undeveloped with the exception of a few locations, Windigo in the southwest and Rock Harbor in the northeast (Fig. 1). Waterbodies must then be accessed by foot or by Park Service boat. Few systematic entomologists have visited ISRO due to the expected depauperate nature of the fauna, though some species that currently live on the island will be of interest since they represent relict populations at the southernmost edge of their range. Another reason for studying aquatic insects at ISRO is to understand which species are capable of colonizing the island. Important questions include what species traits allowed them to colonize successfully, and potentially what sources and routes were involved in the colonization.

Ephemeroptera (mayflies), Plecoptera (stoneflies), and Trichoptera (caddisflies) (EPT species) are environmentally sensitive aquatic insects that are routinely used in monitoring of water quality. Their taxonomy and distribution are relatively well known in the Midwest (Burks 1953, DeWalt et al. 2005, DeWalt et al. 2012, DeWalt and Grubbs 2011, Frison 1935, Grubbs et al. 2013, Houghton 2012, Randolph and McCafferty 1998, Ross 1944). This makes EPT an appropriate target for inventories on ISRO.

The EPT species currently living on ISRO most certainly arrived through one or more of several means: direct flight, drifting with debris, as stowaways on boats, or were already present in the lake. Sources of colonization are streams and lakes along the shoreline of Lake Superior in Michigan, Minnesota, Ontario, and Wisconsin. We are assuming that most species would have flown to the island from mainland shoreline sources and that smaller insect species would arrive and establish in greater frequency than larger ones.

Recent work by DeWalt and colleagues has created a 200,000 record EPT specimen dataset within a seven state area of the Midwest, USA. These data have recently been used to model the historical distributions of stoneflies (Cao et al. 2013) and through the 21^st^ century (DeWalt, unpubl. data). This data set, some recently published records (Houghton 2012), and unpublished data (Klubertanz, pers. comm.) may be used as a tool to build a regional species pool for comparison with ISRO.

The results of our effort to document the EPT species inhabiting the main island of ISRO during 2013 are presented. We hypothesize that the number of EPT species on ISRO is less than that found on the mainland surrounding Lake Superior. In addition, we hypothesize that the size of adult EPT species present on ISRO is smaller than that found on the mainland, suggesting that smaller species are more likely to colonize the island from mainland sources, presumably using prevailing winds.

## Methods

*Inventory*. Inventory of EPT taxa took place over two four-day forays in June and July 2013 (Table 1). Two areas of the island were investigated near access points for ferry service. Our June efforts were concentrated on Lake Superior shorelines and small streams near Windigo in southwestern ISRO (sites 1–9, Table 1, Fig. 1). July efforts concentrated on the same habitats from Rock Harbor in northeastern ISRO to the west end of Moskey Basin (sites 10-21).

Sampling of EPT was conducted in the same manner at each site and continued until no apparently novel taxa were found at a site. Collection of adults was prioritized since species level identification is most straightforward in this life stage. One of the most useful sampling devises for adult EPT ISRO was the beating sheet. The sheet was placed under vegetation at streamside or lakeside and adults dislodged to the sheet. This method was particularly effective when air temperatures were cool, limiting flight of insects after disturbance. Warmer conditions necessitated the use of an aerial sweepnet. Immature EPT were collected using a rectangular dipnet and by handpicking from substrates. The accumulated debris were examined using a white plastic tray and stream water. All EPT specimens were fixed in 95% EtOH. Non-target taxa were released after sorting. The use of ultraviolet light traps was not possible during either of the two visits due to the low early evening air temperatures.

Specimens were identified to the lowest possible taxonomic level, using current literature, and accessioned into the INHS Insect Collection (INHS-IC). These data are available from the INHS-IC database (http://inhsinsectcollection.speciesfile.org/InsectCollection.aspx). Raw specimen data in the form of an Excel comma delimited file are attached as supplementary data.

The relationship between EPT species richness and the number of specimens collected per site was investigated using simple linear regression. This analysis was conducted on untransformed data using VassarStats (Lowry 2015) an internet based statistical package. Data from three samples (sites 12–14 of Table 1) were excluded from this analysis because they were considered incomplete.

*Comparison of ISRO EPT to Mainland.* The mainland list of EPT species was compiled from specimen records whose locations included the Lake Superior border counties of Michigan, Minnesota, and Wisconsin and streams that drained into Lake Superior from Ontario west of -84.3° longitude and south of a line delimited by 49.3° latitude. Specimen data were pulled from the INHS-IC database, several other databases compiled by the senior author from 25 region museums, a regional treatment of mayflies (Randolph and McCafferty 1998), additional mayfly records (T. Klubertanz unpubl. data), and other recent literature (Houghton 2012, Blahnik and Holzenthal 2014, Sun and McCafferty 2008). Some of these data are unpublished, so the species list for the mainland is withheld at the owner’s request. The ISRO list was compared directly to the mainland list.

*Size of ISRO EPT Species Versus Mainland.* Size of specimens was gathered from the literature, often from original species descriptions. Most useful was the Biodiversity Heritage Library, which has made access to older literature efficient. The measure of size varied greatly between sources. Forewing length was preferred, but often body length was the only measure presented. In some of the oldest literature (e.g., Walker 1852), measurements were provided in “lines”. There is no accepted scale for conversion of lines to mm, but a conversion of British lines to 2.12 mm has been offered through http://www.convertunits.com/from/line/to/mm. We have applied this conversion to all line measurements and the resultant sizes agree with congeners measured in mm. For all but the largest of EPT species, body length appeared to be a suitable approximation of forewing length. In some instances, no adult measures were available, so length of mature larvae was recorded or measures from species in the same genus were used. Ranges of sizes were often presented in literature sources and were recorded as both minimum and maximum size. Admittedly, some error exists in the sizes recorded, but this appears to be the best that can be done without actually measuring replicates of several hundred species. Literature sources and the type of measure were recorded for all species. Those who wish to use the data set may request a copy from the senior author.

Since we were only assured of a minimum size across the entire data set, this was the measure used for comparative purposes. Both integer and decimal values were present in the literature, so all were converted to the integer form of the value to simplify analysis. Frequency histograms with size classes from 1 to 34 mm were compared for mainland and ISRO species. A Kruskal-Wallis k=3 analysis of ranked data was conducted to compare sizes of orders of EPT on the mainland and on the ISRO (Sokal and Rohlf 1981). In addition, a Mann-Whitney U-test was conducted on mainland versus ISRO adult size for each order (Sokal and Rohlf 1981). All tests of significance were run using Lowry (2015).

## Results

**Figure 2.**
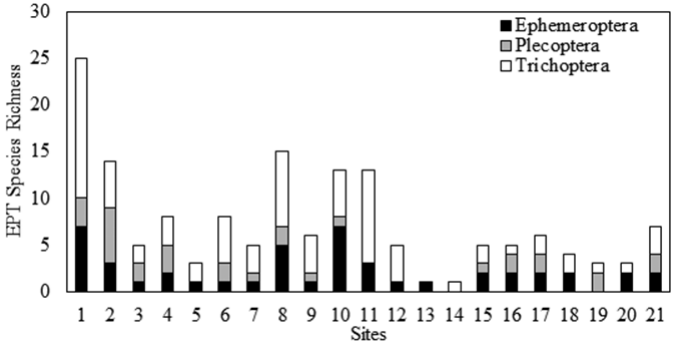
Ephemeroptera, Plecoptera, and Trichoptera species richness recovered from Isle Royale National Park sites during 2013. Site numbers correspond to those in Table 1.

**Figure 3.**
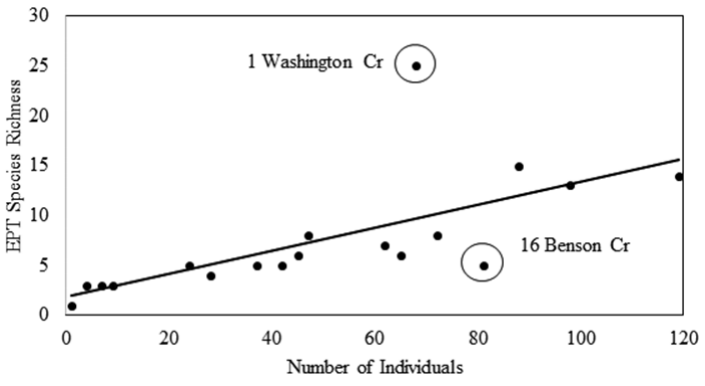
Relationship of Ephemeroptera, Plecoptera, and Trichoptera species richness to the number of individuals found at 18 sites where full samples were taken on Isle Royale National Park, 2013. Circled points indicate sites that had higher and lower than predicted richness. Diagonal is line-of-best-fit.

**Figure 4.**
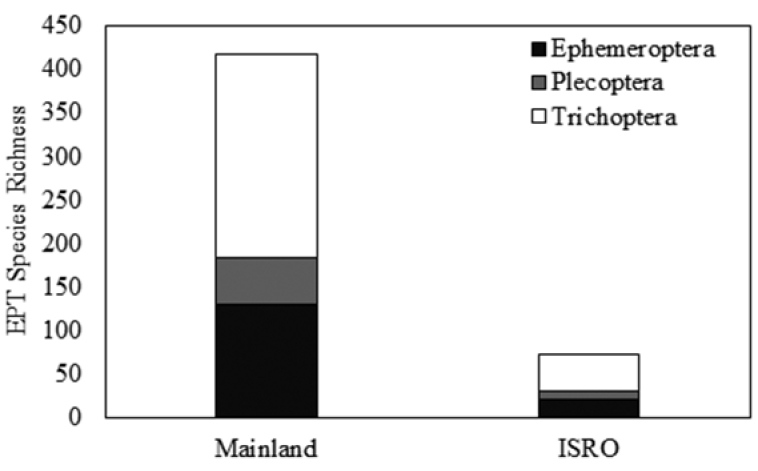
Comparison of Ephemeroptera, Plecoptera, and Trichoptera species richness for mainland around Lake Superior versus that found on Isle Royale National Park sites during 2013.

*ISRO EPT Richness and Comparison to Mainland.* Twenty-nine samples were collected from ISRO during 2013, representing 21 locations from opposite ends of the island (Fig. 1, Table 1). These samples produced 983 specimens representing 73 species of EPT (Table 2). The vast majority of EPT species were caddisflies, contributing 42 of the 73 species reported. Mayflies contributed 22 species, while stoneflies contributed only nine. Site EPT richness varied dramatically (Fig. 2). The EPT species richness for completely sampled sites was a linear function of the number of individuals found at the site (simple linear regression, R^2^=0.45, p=0.002, n=18, Fig. 3). Washington Creek departed greatly from the line-of-best fit (Site 1 of Table 1, Fig. 3). This 5 m wide trout stream is much more diverse compared to other streams sampled during this project, supporting 25 EPT species from a relatively modest number of specimens. The similarly sized Grace Creek (Site 8, Fig. 2) produced only 15 EPT species. Other relatively diverse sites were Lake Superior shorelines at Huginnin Cove (Site 2), Daisy Farm Campground (Site 10), and at Moskey Bay Campground (Site 11). Benson Creek (Site 16 of Table 1) under performed versus predicted richness. This 2 m wide stream produced just five EPT species including two mayflies, two stoneflies, and one caddisfly (Table 2, Fig. 2). Mainland richness was much higher than that found on ISRO, confirming our hypothesis. This trend held for each order sampled, with 417 EPT species being recorded from mainland specimen and literature sources (Fig. 4).

**Table 2.** Ephemeroptera, Plecoptera, and Trichoptera (EPT taxa) recovered from Isle Royale National Park locations during 2013. The site number is the same as used in Table 1. Genera were added to richness measures if no species level identification was available for a given location. ms = medium stream (3–10 m wide), l = lakeshore, sl = small lakeshore, ss = small stream, and (1–2 m wide). *Incomplete sampled locations. **Indicates new state record.

Site Number→ Taxon ↓	1	2	3	4	5	6	7	8	9	10	11	12*	13*	14*	15	16	17	18	19	20	21	∑
**Ephemeroptera-mayflies**																						
**Baetidae-small minnow mayflies**																						
*Acerpenna macdunnoughi* (Ide, 1937)	1	0	0	0	0	0	0	14	0	0	0	0	0	0	0	0	0	0	0	0	0	15
*Baetis brunneicolor* McDunnough, 1925	1	0	0	0	0	0	0	1	0	0	0	0	0	0	15	36	12	0	0	0	0	65
*Baetis bundyae* Lehmkuhl, 1973**	0	0	7	1	0	0	0	0	0	0	0	0	0	0	0	0	0	0	0	0	0	8
*Baetis flavistriga* McDunnough, 1921	1	0	0	0	0	0	0	4	0	1	0	0	0	0	0	0	0	0	0	0	10	16
*Baetis tricaudatus* Dodds, 1923	0	0	0	0	0	0	0	0	21	0	0	0	0	0	0	0	0	0	0	0	0	21
*Callibaetis ferrugineus* (Walsh, 1862)	0	0	0	0	0	0	0	0	0	0	0	0	2	0	0	0	0	0	0	0	0	2
*Neocloeon triangulifer* (McDunnough, 1931)	0	0	0	0	0	0	0	0	0	0	0	0	0	0	0	0	0	16	0	0	0	16
*Neocloeon* sp.	0	0	0	0	0	0	0	0	0	0	0	1	0	0	0	0	0	0	0	0	0	1
**Caenidae-small square-gilled mayflies**																						
*Caenis latipennis* Banks, 1907	0	0	0	0	0	0	0	0	0	0	0	0	0	0	0	0	0	0	0	1	0	1
**Ephemerellidae-spiny crawler mayflies**																						
*Eurylophella bicolor* (Clemens, 1913)	0	0	0	0	0	0	0	0	0	22	0	0	0	0	0	0	0	0	0	0	0	22
*Eurylophella funeralis* (McDunnough, 1925)	0	0	0	0	0	5	0	0	0	0	0	0	0	0	0	0	0	0	0	0	0	5
*Eurylophella temporalis* (McDunnough, 1924)	0	0	0	0	0	0	0	0	0	0	1	0	0	0	0	0	0	0	0	0	0	1
**Ephemeridae-burrowing mayflies**																						
*Ephemera simulans* Walker, 1853	0	0	0	0	0	0	0	0	0	4	0	0	0	0	0	0	0	0	0	0	0	4
*Hexagenia limbata* (Serville, 1829)	0	0	0	0	0	0	0	0	0	0	3	0	0	0	0	0	0	0	0	0	0	3
**Heptageniidae-flat-headed mayflies**																						
*Heptagenia pulla* (Clemens, 1913)	0	1	0	0	0	0	0	0	0	1	0	0	0	0	0	0	0	0	0	0	0	2
*Leucrocuta* sp.	0	0	0	0	0	0	0	0	0	0	1	0	0	0	0	0	0	0	0	0	0	1
*Maccaffertium vicarium* (Walker, 1853)	7	0	0	0	0	0	0	4	0	0	0	0	0	0	0	0	0	0	0	0	0	11
*Nixe* sp.	1	0	0	0	0	0	0	0	0	2	0	0	0	0	0	0	0	0	0	0	0	3
*Stenonema femoratum* (Say, 1823)	0	0	0	0	0	0	0	0	0	1	0	0	0	0	0	0	0	0	0	0	0	1
**Leptophlebiidae-prong-gilled mayflies**																						
*Leptophlebia* sp.	0	1	0	0	0	0	0	0	0	1	0	0	0	0	0	0	0	0	0	0	4	6
*Paraleptophlebia adoptiva* (McDunnough, 1929)	6	0	0	0	0	0	0	11	0	0	0	0	0	0	0	0	0	0	0	0	0	17
*Paraleptophlebia praepedita* (Eaton, 1884)	0	0	0	0	0	0	0	0	0	0	0	0	0	0	2	1	4	7	0	0	0	14
**Siphlonuridae-primitive minnow mayflies**																						
*Siphlonurus phyllis* McDunnough, 1923**	1	5	0	22	4	0	1	0	0	0	0	0	0	0	0	0	0	0	0	2	0	35
**Plecoptera-stoneflies**																						
**Capniidae-winter stoneflies**																						
*Capnia vernalis* (Newport, 1851)	0	6	0	0	0	0	0	0	0	0	0	0	0	0	0	0	0	0	0	0	0	6
*Paracapnia angulata* Hanson, 1942	0	77	0	0	0	0	0	0	0	0	0	0	0	0	0	0	0	0	0	0	0	77
**Chloroperlidae-sallflies**																						
*Haploperla brevis* (Banks, 1895)	5	1	1	0	0	0	0	13	0	51	0	0	0	0	0	0	0	0	1	0	25	97
**Leuctridae-needleflies**																						
*Leuctra ferruginea* (Walker, 1852)	4	0	0	12	0	23	0	1	1	0	0	0	0	0	0	7	18	0	0	0	0	66
**Nemouridae-forest stoneflies**																						
*Amphinemura palmeni* (Koponen, 1917)	0	0	0	1	0	0	0	0	0	0	0	0	0	0	12	35	21	0	6	0	0	75
*Nemoura trispinosa* Claassen, 1923	0	7	17	2	0	17	7	0	0	0	0	0	0	0	0	0	0	0	0	0	0	50
**Perlodidae-spring stoneflies**																						
*Arcynopteryx dichroa* (McLachlan, 1872)	0	7	0	0	0	0	0	0	0	0	0	0	0	0	0	0	0	0	0	0	0	7
*Isoperla bilineata* (Say, 1823)	0	7	0	0	0	0	0	0	0	0	0	0	0	0	0	0	0	0	0	0	1	8
*Isoperla transmarina* (Newman, 1838)	1	0	0	0	0	0	0	0	0	0	0	0	0	0	0	0	0	0	0	0	0	1
**Trichoptera-caddisflies**																						
**Apataniidae--early smoky wing sedges**																						
*Apatania zonella* Zetterstedt, 1840**	0	0	0	0	0	0	0	0	0	2	0	0	0	0	0	0	0	0	0	0	15	17
**Dipseudopsidae-bristle sedge caddisfly**																						
*Phylocentropus placidus* Banks, 1905	0	0	0	0	0	0	0	0	0	0	4	0	0	0	0	0	0	0	0	0	0	4
**Glossosomatidae-saddlecase caddisflies**																						
*Glossosoma intermedium* Klapálek, 1892	3	1	0	0	0	0	0	0	4	0	0	0	0	0	0	0	0	0	0	0	1	9
*Glossosoma nigrior* Banks, 1911	0	0	0	0	0	0	0	11	0	0	0	0	0	0	0	0	0	0	0	0	0	11
**Helicopsychidae-snailcase caddisflies**																						
*Helicopsyche borealis* Hagen, 1861	0	0	0	0	0	0	0	0	0	3	22	0	0	0	0	0	0	0	0	0	0	25
**Hydropsychidae-net-spinning caddisfly**	0	0	0	0	0	0	0	0	0	0	0	0	0	0	0	0	0	0	0	0	0	0
*Cheumatopsyche* sp.	1	0	0	0	0	0	0	12	0	0	0	0	0	0	0	0	0	0	0	0	0	13
*Hydropsyche alhedra* Ross, 1939	1	0	0	0	0	0	0	0	0	0	0	0	0	0	0	0	0	0	0	0	0	1
*Hydropsyche alternans* Walker, 1852	0	0	0	0	0	0	0	0	0	0	0	0	0	0	0	0	0	0	0	0	6	6
*Hydropsyche betteni* Ross, 1938	0	0	0	0	0	0	0	2	0	0	0	0	0	0	0	0	0	0	0	0	0	2
*Hydropsyche bronta* Ross, 1938	0	0	0	0	0	0	0	0	0	0	0	0	0	0	0	0	0	0	0	0	0	0
*Hydropsyche slossonae* Banks, 1905	1	0	0	0	0	0	0	0	0	0	0	0	0	0	0	0	0	0	0	0	0	1
*Hydropsyche morosa* group	6	0	0	0	0	0	0	3	0	0	0	0	0	0	0	0	0	0	0	0	0	9
*Parapsyche apicalis* (Banks, 1908)	0	0	0	0	0	7	0	0	13	0	0	0	0	0	0	0	0	0	0	0	0	20
**Hydroptilidae-microcaddisflies**																						
*Hydroptila* sp.	1	0	0	0	0	0	0	2	0	0	0	0	0	0	0	0	0	0	0	0	0	3
*Ochrotrichia* sp.	0	0	0	0	0	0	0	0	0	0	0	0	0	0	0	0	0	0	0	0	0	0
*Oxyethira* sp.	1	0	0	0	0	0	0	0	0	0	0	0	0	0	0	0	0	0	0	0	0	1
**Lepidostomatidae-lepidostomatid casemaking caddisflies**																						
*Lepidostoma togatum* Hagen, 1861	0	0	0	0	0	0	0	0	0	7	1	0	0	0	0	0	0	0	0	0	0	8
*Lepidostoma* sp.	0	1	9	5	0	3	0	0	0	0	0	0	0	0	0	2	1	0	0	0	0	21
**Leptoceridae-longhorned caddisflies**																						
*Mystacides interjecta* (Banks, 1914)	0	0	0	0	0	0	0	0	0	0	0	7	0	0	0	0	0	0	0	0	0	7
*Mystacides sepulchralis* Walker, 1852	0	0	0	0	0	0	0	0	0	0	19	0	0	0	0	0	0	0	0	0	0	19
*Oecetis avara* Banks, 1895	7	0	0	0	0	0	0	0	0	0	0	0	0	0	0	0	0	0	0	0	0	7
*Oecetis cinerascens* Hagen, 1861	0	0	0	0	0	0	0	0	0	0	0	2	0	0	0	0	0	0	0	0	0	2
*Oecetis osteni* Milne, 1934	0	0	0	0	0	0	0	0	0	0	0	1	0	0	0	0	0	0	0	0	0	1
*Oecetis* sp.	1	0	0	0	0	0	0	0	0	0	0	0	0	0	0	0	0	0	0	0	0	1
*Triaenodes injustus* Hagen, 1861																						
*Triaenodes nox* Ross, 1941	0	0	0	0	0	0	0	0	0	0	0	0	0	1	0	0	0	0	0	0	0	1
**Limnephilidae-northern casemaking caddisflies**	0	0	0	0	0	0	0	0	0	0	0	0	0	0	0	0	0	0	0	1	0	1
*Anabolia consocia* (Walker, 1852)	0	0	0	0	0	0	0	0	0	0	0	0	0	0	2	0	0	0	0	0	0	2
*Anabolia sordida* Hagen, 1861	0	0	0	1	0	0	0	0	0	0	0	0	0	0	0	0	0	0	0	0	0	1
*Anabolia* sp.	1	0	0	0	0	0	0	0	0	0	2	1	0	0	0	0	0	2	0	0	0	6
*Hesperophylax designatus* Walker, 1852	0	0	0	0	0	6	5	0	0	0	0	0	0	0	0	0	0	0	0	0	0	11
*Ironoquia parvula* Banks, 1900**	0	0	0	0	2	0	0	0	0	0	0	0	0	0	0	0	0	0	0	0	0	2
*Limnephilus moestus* Banks, 1908	0	0	0	0	0	0	0	0	0	0	0	0	0	0	0	0	0	0	2	0	0	2
*Limnephilus parvulus* Banks, 1905	0	2	0	0	0	0	0	0	0	0	0	0	0	0	0	0	0	0	0	0	0	2
*Limnephilus rhombicus* Linnaeus, 1758	4	0	0	0	1	0	0	0	0	0	0	0	0	0	0	0	0	0	0	0	0	5
*Limnephilus* sp.	0	0	0	0	0	0	0	0	0	0	1	0	0	0	0	0	0	0	0	0	0	1
*Nemotaulius hostilis* Hagen, 1873	0	0	0	0	0	0	0	0	0	1	0	0	0	0	0	0	0	0	0	0	0	1
*Platycentropus radiatus* Say, 1824	0	0	0	0	0	0	0	1	0	0	0	0	0	0	0	0	0	0	0	0	0	1
**Molannidae-hoodcase making caddisflies**																						
*Molanna blenda* Sibley, 1926	3	0	0	0	0	0	0	0	0	0	0	0	0	0	0	0	0	3	0	0	0	6
*Molanna flavicornis* Banks, 1914	0	0	0	0	0	0	0	0	0	0	8	0	0	0	0	0	0	0	0	0	0	8
*Molanna* sp.	0	0	0	0	0	0	0	0	0	0	3	0	0	0	0	0	0	0	0	0	0	3
**Philopotamidae-fingernet caddisfly**																						
*Chimarra* sp.	1	0	0	0	0	0	0	0	0	0	0	0	0	0	0	0	0	0	0	0	0	1
*Dolophilodes distincta* (Walker, 1852)	0	0	0	0	0	0	0	0	1	0	0	0	0	0	0	0	9	0	0	0	0	10
**Phryganeidae-giant caddisflies**																						
*Agrypnia straminea* Hagen, 1873	0	0	0	0	0	0	0	0	0	0	6	0	0	0	0	0	0	0	0	0	0	6
**Polycentropodidae-trumpetnet caddisflies**																						
*Nyctiophylax* sp.	2	0	0	0	0	0	0	0	0	0	0	0	0	0	0	0	0	0	0	0	0	2
*Plectrocnemia cinerea* Hagen, 1861	0	0	0	0	0	0	0	0	0	0	1	0	0	0	0	0	0	0	0	0	0	1
*Polycentropus* sp.	0	2	0	3	0	1	0	0	0	0	0	0	0	0	0	0	0	0	0	0	0	6
**Psychomyiidae-trumpetnet caddisflies**																						
*Psychomyia flavida* Hagen, 1861	1	0	0	0	0	0	0	0	0	0	0	0	0	0	0	0	0	0	0	0	0	1
**Rhyacophilidae-predatory caddisflies**																						
*Rhyacophila vibox* Milne, 1936	2	0	0	0	0	0	2	6	0	0	0	0	0	0	0	0	0	0	0	0	0	10
**Thremmatidae-thremmatid stonecase makers**																						
*Neophylax concinnus* McLachlan, 1871	4	1	8	0	0	10	9	3	5	2	0	0	0	0	6	0	0	0	0	0	0	48
∑	68	119	42	47	7	72	24	88	45	98	72	12	2	1	37	81	65	28	9	4	62	983
Ephemeroptera	7	3	1	2	1	1	1	5	1	7	3	1	1	0	2	2	2	2	0	2	2	22
Plecoptera	3	6	2	3	0	2	1	2	1	1	0	0	0	0	1	2	2	0	2	0	2	9
Trichoptera	15	5	2	3	2	5	3	8	4	5	10	4	0	1	2	1	2	2	1	1	3	42
EPT Taxa	25	14	5	8	3	8	5	15	6	13	13	5	1	1	5	5	6	4	3	3	7	73
Sites	1	2	3	4	5	6	7	8	9	10	11	12	13	14	15	16	17	18	19	20	21	
Waterbody size/type	ms	l	ss	ss	ss	ss	ss	ms	ss	l	l	sl	ms	ss	ss	ss	ss	ss	ss	ms	l	
Used in species richness estimation/averages	y	y	y	y	y	y	y	y	y	y	y	n	n	n	y	y	y	y	y	y	y	

**Figure 5.**
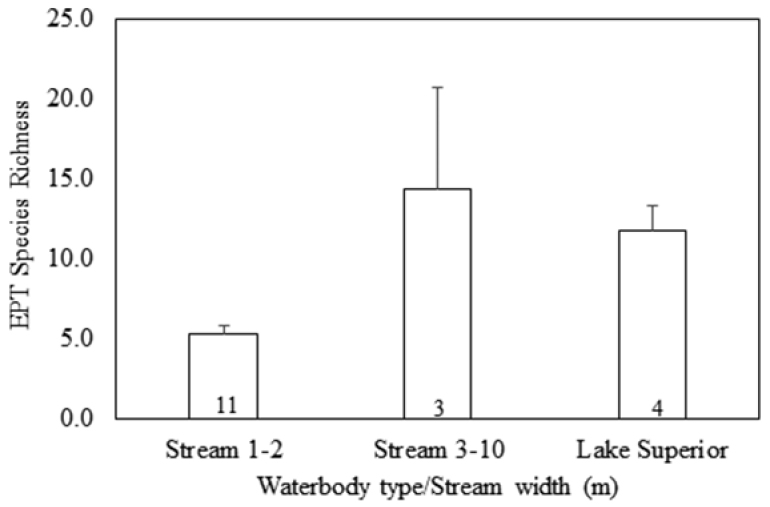
Mean site Ephemeroptera, Plecoptera, and Trichoptera species richness by waterbody type and stream size. Sites represented by incomplete samples excluded. Numbers in columns indicate number of sites. Error bars indicated standard error of the mean.

Species richness was predictable in relation to waterbody type and stream size. Streams 1–2 m wide supported a limited EPT fauna, averaging 5.3 species with narrow variability (Fig. 5). Larger streams supported many more species with much higher variation. Lake Superior shorelines, including areas open to the fetch of the lake and those in large protected bays, produced on average 10 EPT species, with relatively low variability.

**Figure 6.**
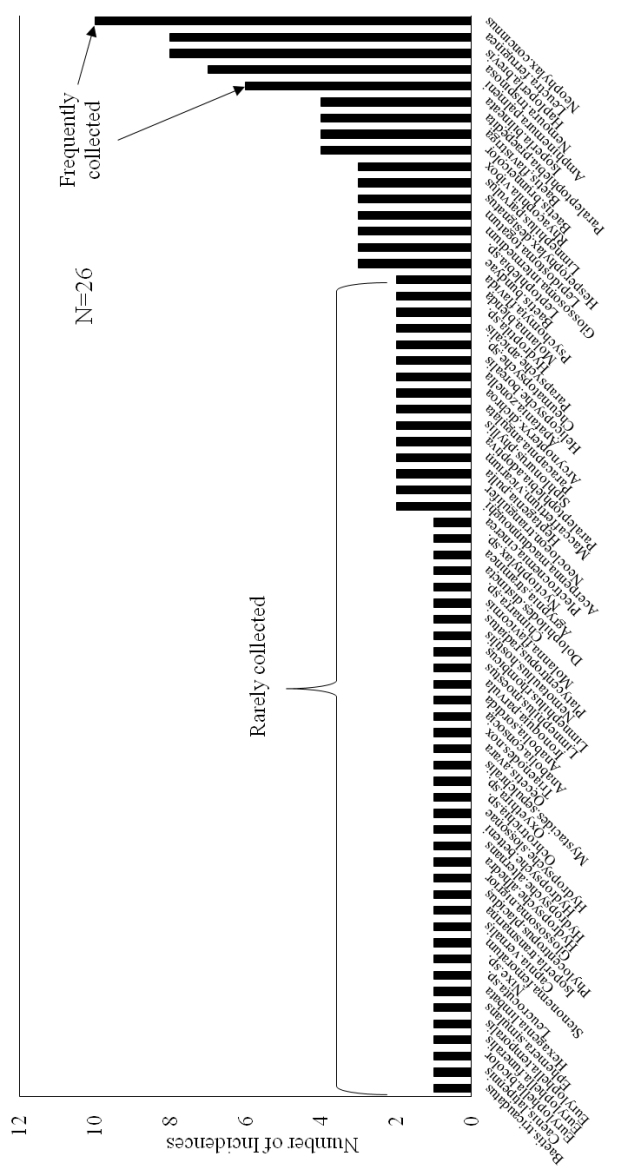
Number of sample incidences for Ephemeroptera, Plecoptera, and Trichoptera species on Isle Royale National Park, 2013. Incomplete samples excluded.

Most EPT species found on ISRO were rarely encountered, 51 of them being present in only one or two samples of 26 complete samples (Fig. 6). A relatively few species may be considered common on ISRO since they were found in >4 samples. Among these were five species: the caddisfly *Neophylax concinnus* McLachlan, 1871 and the stoneflies *Amphinemura palmeni* (Koponen, 1917), *Leuctra ferruginea* (Walker, 1852), *Nemoura trispinosa* Claassen, 1923, and *Haploperla brevis* (Banks, 1895).

**Figure 7.**
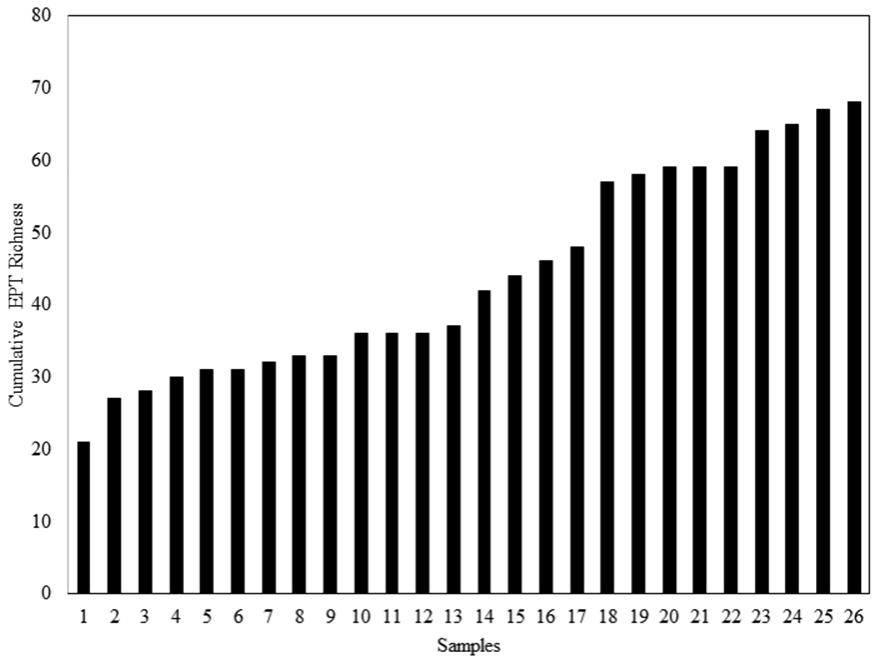
Cumulative Ephemeroptera, Plecoptera, and Trichoptera species richness generated from 26 complete samples collected from Isle Royale National Park, 2013.

We were unable to produce reliable predictions of EPT species richness for ISRO with the number of complete samples at hand. Cumulative richness from complete samples yielded 68 species (Fig. 7). Five additional species from three incomplete samples bring the total to 73 species.

**Figure 8.**
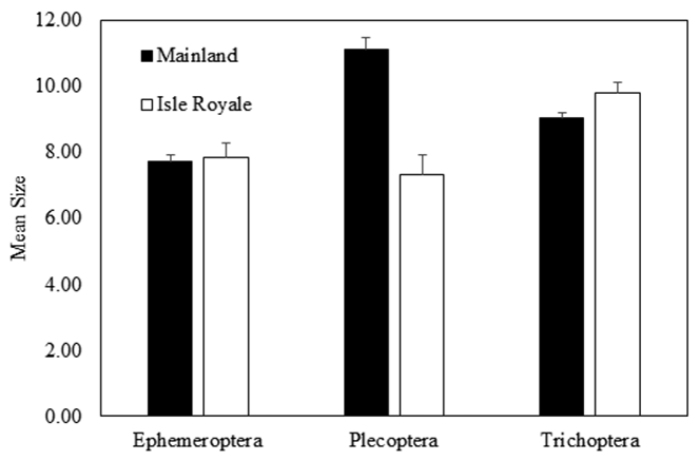
Mean and standard error of minimum size of Ephemeroptera, Plecoptera, and Trichoptera for species inhabiting the Lake Superior mainland and species sampled from Isle Royale National Park lakeshore and streams.

**Figure 9.**
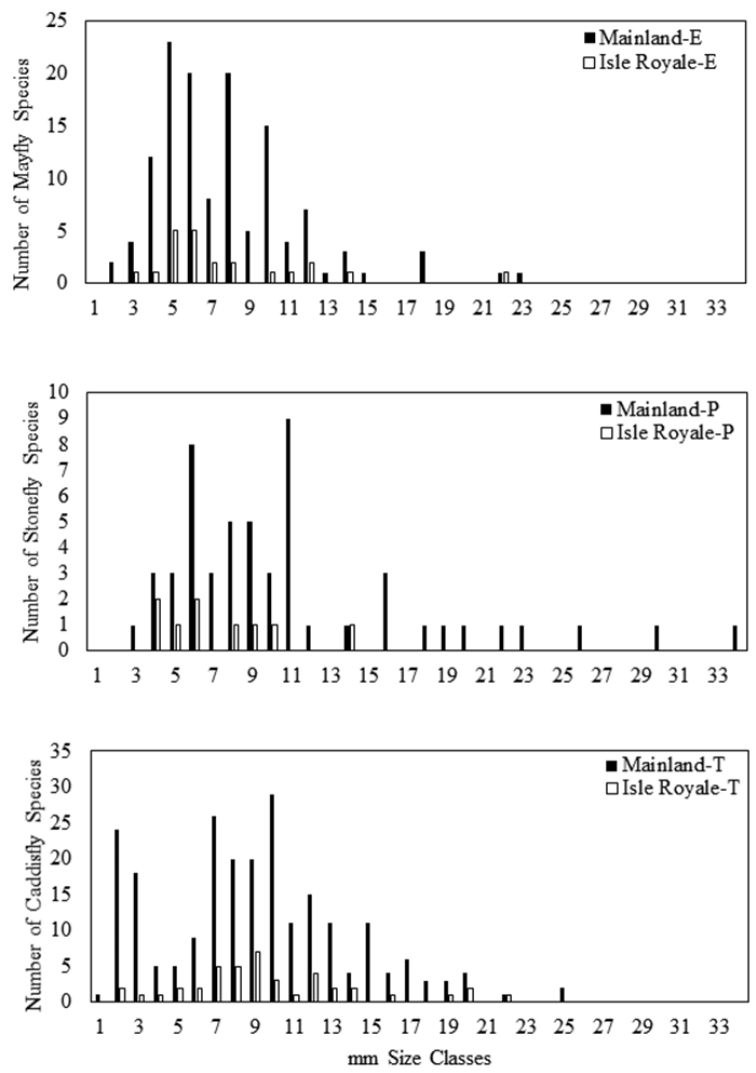
Size frequency histogram of Ephemeroptera (**E**), Plecoptera (**P**), and Trichoptera (**T**) species inhabiting Lake Superior mainland and species sampled from Isle Royale National Park lakeshore and streams.

*Size of ISRO EPT Species Versus Mainland.* Mainland EPT were significantly different in size across orders (Kruskal-Wallis, H=13.9, df=2, p=0.0009), with stoneflies having the largest average size at 11.13 mm (Fig. 8). Alternatively, EPT size on ISRO was not significantly different across orders, but the margin was close with the mean size of caddisflies being somewhat larger than other orders (Kruskal-Wallis, H=5.7, df=2, p=0.059) (Fig. 8). With respect to comparisons between sources, mayfly species size between the mainland and ISRO was not significantly different (Mann-Whitney U-test, U=1403.0, P(1)=0.44, P(2)=0.89). A frequency histogram demonstrates that sizes of mayflies overlapped greatly for mainland and ISRO sources (Fig. 9). Stonefly species were significantly smaller on ISRO than they were on the mainland (Mann-Whitney U-test, U=149.5, P(1)=0.034, P(2)=0.067). Mainland stonefly species ranged 3–34 mm in size, while on ISRO, they ranged 4–14 mm (Fig. 9). Caddisfly species size was not significantly different between sources (Mann-Whitney U-test, U=5231.0, P(1)=0.239, P(2)=0.478) with the size distribution of the two sources being nearly identical (Fig. 9).

## Discussion

Aquatic resources on ISRO, with the current state of sampling, support only 17.5% of the EPT species found on the mainland surrounding Lake Superior (Fig. 4). Since the number of species found in only one or two samples is large (Fig. 6) and the accumulation of species has a steep curve (Fig. 7), we assume that this proportion will rise with greater effort. However, we still expect that <50% of the mainland species will be recovered from ISRO. Caddisflies seemed to be the most effective colonizers of the island, their observed richness being nearly 57.6% of the EPT species recorded to date (Fig. 4). On the other hand, stoneflies seem to be ill suited for traversing the distance from the mainland to ISRO. However, those that colonized ISRO often occurred at many sites (Fig. 6) and often in high abundance.

Small streams on ISRO supported low EPT species richness, while the number of species climbed dramatically in larger streams (Fig. 5). It is probable that many of these small streams freeze into the streambed during the winter, leaving only a few hardy species with egg diapause to overwinter. The larger, species rich streams must continue flowing during winter. Caddisflies and mayflies were most responsible for the increase in species richness of larger streams. Interestingly, Washington and Grace creeks, though being of nearly identical size and gradient, and being geographically close, were quite different in assemblage composition with a Sorensen’s Index of Similarity being only 38%.

The shores of Lake Superior supported a moderate diversity of EPT with low variability in the number of species. The communities varied greatly between cold beaches exposed to the fetch of the lake and the protected bays. Exposed lake shores produced many more stoneflies than protected bays, presumably due to the colder water. Caddisflies and mayflies were much more species rich in the protected bays.

*Body Size as a Predictor of ISRO EPT.* Others have studied the evolution of body size of vertebrates on isolated islands as a function of food quality, island area, and interactions with other species (Boyer and Jetz 2010). We are focusing on size as a factor important only in the initial colonization of aquatic insects to ISRO. We suggest that large species may not colonize ISRO successfully and that small species would have an advantage since they could use updrafts from Minnesota, Ontario, or the Keweenaw Peninsula of Michigan to reach ISRO. We have found that the assemblage of stoneflies on ISRO support this hypothesis since they were significantly smaller than on the mainland (Fig. 9). The two largest stonefly species on ISRO, *Isoperla bilineata* (Say, 1824) (9–9.5 mm) and *Arcynopteryx dichroa* (McLachlan, 1872) (14–15 mm), occurred in the lake and presumably have used it to colonize the island. Stoneflies are often considered poor fliers (Stewart and Stark 2002), although there is little direct evidence for this. Malmqvist (2000) found that wing length was positively related to range size and that species with short wings were most likely to be rare and isolated on the landscape. One the mainland, 10–20 large species in the families Perlodidae, Perlidae, and Pteronarcyidae may be present in the same stream. Stoll et al. (2014) found that the presence of fish in the regional species pool was a most important determiner of colonization of restored stream reaches. We suggest that the distance and disruption of normal habitat, e.g. the lake, for larval and adult stonefly species limits most large species from reaching ISRO. One mechanism for limiting flight of large stoneflies is that they fly with the body inclined at a 25–45° angle, conferring considerable drag during flight (DeWalt pers. obs.). The complete absence of truly large stoneflies on ISRO suggests that larger stonefly species do not have the energy reserves or aerodynamics to fly or draft the long distances from the mainland to ISRO.

Mayflies and caddisflies do not support the hypothesis that smaller species are more likely to colonize ISRO (Figs 8, 9). Mayfly wing length has been demonstrated to be positively related to range size, a trait that would increase the possibility of them flying from mainland to ISRO (Malmqvist 2000). Mayflies and caddisflies are generally thought of as stronger fliers than stoneflies. Indeed, anecdotal evidence from weather radar supports the idea that large, burrowing mayflies in the genus *Hexagenia* (Ephemeridae) fly considerable distances equivalent to that that isolates ISRO from the mainland (Washington Post 2014). The body axis orientation of mayflies and caddisflies is more horizontal during flight; presumably, more power is transferred to forward motion without the drag that stoneflies endure. In addition, an unknown number of mayfly and caddisfly species included in the ISRO taxa list certainly occur within Lake Superior. This would make the habitat from mainland to ISRO more continuous and allow more species of both orders to reach the island. Of course, examination of many more species traits is necessary to determine which factors are most important to the postglacial recolonization of ISRO by all three groups.

*Taxa of Significance.* Most species reported herein have never been reported in the literature from the ISRO and represent a leap in knowledge of the species of aquatic insects that inhabit the park. Some species were of particular interest because of their rarity in the region, their being new state records, or because they have been known in the region under different names until recently. We present an annotated list of those species.

### Ephemeroptera

*Acerpenna macdunnoughi* (Ide, 1937). Several records exist for small streams in Marquette County, Michigan (Randolph and McCafferty 1998) and nowhere else in the state. It was found in both Washington (Site 1) and Grace creeks (Site 8), the latter in abundance.

*Baetis bundyae* Lehmkuhl, 1973. This boreal/tundra species has not been reported from Michigan before (Randolph and McCafferty 1998), but is known from nearby northeastern Minnesota (Lager et al. 1982). This coldwater species was found in a two locations: Huginnin Creek at Huginnin Cove (Site 3) and at a nearby unnamed tributary to Lake Superior (Site 4). This area is kept cold by the lake breezes, producing a southern refuge for the species.

*Callibaetis ferrugineus* (Walsh, 1862). This is the first record of the species for upper Michigan (Randolph and McCafferty 1998). Two nymphs were taken from the outlet of Lake Richie (Site 12) along the Indian Portage Trail.

*Neocloeon triangulifer* (McDunnough, 1931). This species, under the name *Centroptilum triangulifer* (McDunnough, 1931), was only recently added to the Michigan mayfly list, the new records being from Baraga and Marquette counties in the Upper Peninsula (McCafferty 2009). Jacobus and Wiersema (2014) recently moved this species to *Neocloeon*. A large population was taken from the flooded mouth of a small tributary to Lake Superior near Moskey Basin Campground (Site 18).

*Paraleptophlebia praepedita* (Eaton, 1884). This has been rarely collected from Michigan, and only in the lower third of the state (Randolph and McCafferty 1998). It was taken from several tributaries to Moskey Basin (Sites 15-18). This is the first record of the species in northern Michigan.

*Siphlonurus phyllis* McDunnough, 1923. This species has never been reported from the state (Randolph and McCafferty 1998). Its presence represents a new state record.

### Plecoptera

*Capnia vernalis* (Newport, 1851). This species is rare in the region. It was found only at the Lake Superior shoreline at Huginnin Cove (Site 2).

*Amphinemura palmeni* (Koponen, 1917). It is not surprising that this species was found on ISRO. We have listed it here to call attention to a relatively recent synonymy that has occurred. Probably hundreds of specimens exist in North American collections using the name *Amphinemura linda* (Ricker, 1952), a junior synonym (Boumans and Baumann 2012). This is apparently the only *Amphinemura* on the island and was found at five small streams (Sites 4, 15–17, 19).

*Arcynopteryx dichroa* (McLachlan, 1872). This Holarctic species is another rarity, being known only from the shores of Lake Superior in the region (Grubbs and Bright 2001). Until recently it was known as *Arcynopteryx compacta* (McLachlan, 1872), but all Nearctic specimens under that name are now referable to *Arcynopteryx dichroa* (Teslenko 2012).

### Trichoptera

*Apatania zonella* Zetterstedt, 1840. This is a new state record for Michigan. Leonard and Leonard (1949) did not report it for the state, but it has been collected from Lake Superior shores in nearby Minnesota. We collected it from shoreline samples at Daisy Farm Campground (Site 10) and Rock Harbor (Site 21).

*Ironoquia parvula* Banks, 1900. This too is a new state record for Michigan. We collected two of the distinctive (Flint 1960) larvae from a small, white cedar swamp (site 5).

## Supplementary materials

Supplementary material 1 Table S1. Raw specimen data in the form of an Excel comma delimited file Data type: occurence This dataset is made available under the Open Database License (http://opendatacommons.org/licenses/odbl/1.0/). The Open Database License (ODbL) is a license agreement intended to allow users to freely share, modify, and use this Dataset while maintaining this same freedom for others, provided that the original source and author(s) are credited.R. Edward DeWalt, Eric. J. South
